# Use of the Serum Level of Cholinesterase as a Prognostic Marker of Nonfatal Clinical Outcomes in Patients Hospitalized with Acute Exacerbations of Chronic Obstructive Pulmonary Disease

**DOI:** 10.1155/2024/6038771

**Published:** 2024-03-12

**Authors:** Zhixiang Chen, Lei Zha, Bin Hu, Bin Xu, Lin Zuo, Jun Yang, Zhuhua Chu, Lingling Ma, Fangfang Hu

**Affiliations:** ^1^Department of Respiratory and Critical Care Medicine, Wuhu Hospital of Traditional Chinese Medicine, 430 Jiuhua South Road, Wuhu, Anhui Province, China; ^2^Department of Respiratory Medicine, The First Affiliated Hospital of Wannan Medical College (Yijishan Hospital), Wuhu City, Anhui Province, China

## Abstract

**Introduction:**

Acute exacerbation of chronic obstructive pulmonary disease (AECOPD) contributes to a poor prognosis. Reliable biomarkers to predict adverse outcomes during hospitalization are important.

**Aim:**

To investigate the relationship between the serum cholinesterase (ChE) level and adverse clinical outcomes, including hypoxemia severity, hypercapnia, duration of hospital stay (DoHS), and noninvasive ventilation (NIV) requirement, in patients with AECOPD.

**Methods:**

Patients hospitalized with AECOPD in the Wuhu Hospital of Traditional Chinese Medicine between January 2017 and December 2021 were included.

**Results:**

A total of 429 patients were enrolled. The serum ChE level was significantly lower in patients with hypercapnia, who required NIV during hospitalization and who had a DoHS of >10 days, with an oxygenation index < 300. The ChE level was correlated negatively with the C-reactive protein level and neutrophil-to-lymphocyte ratio and correlated positively with the serum albumin level. Multivariate logistic regression analysis indicated that a serum ChE level of ≤4116 U/L (OR = 2.857, 95% CI = 1.46–5.58, *p* = 0.002) was associated significantly with NIV requirement.

**Conclusions:**

The serum ChE level was correlated significantly with complicating severe hypoxemia, hypercapnia, prolonged DoHS, and the need for NIV in patients hospitalized with AECOPD. The serum ChE level is a clinically important risk-stratification biomarker in patients hospitalized with AECOPD.

## 1. Introduction

Chronic obstructive pulmonary disease (COPD) is a chronic inflammatory disease of the lower respiratory tract [1–3]. COPD was the third leading cause of death worldwide in 2020 [4–6]. Acute exacerbation is inevitable during the disease course and is also the most common reason for hospital admission among patients with COPD [7]. Often, acute exacerbation of chronic obstructive pulmonary disease (AECOPD) results in a reduced quality of life, accelerated disease progression, increased healthcare costs, and increased risk of death [8–10]. Risk stratification of AECOPD is crucial for determining clinical management [11, 12], but a unified standard for assessing the severity and progression of AECOPD using a clinical application is lacking. Thus, searching for reliable tools (including biomarkers that can predict an adverse prognosis accurately in hospitalized patients with AECOPD) is extremely important.

The enzyme cholinesterase (ChE) catalyzes the hydrolysis of choline-based esters. ChE is synthesized mainly in hepatocytes and released into blood [13] and is also involved in the regulation of the balance between the proliferation and death, as well as modulation of the adhesion and migration of cells [14]. Recent studies have suggested that the serum level of ChE is closely associated with inflammation, injury, infections, and malnutrition [15]. The serum ChE level can be measured conveniently through noninvasive blood testing. Hence, it is used widely as a significant predictor for clinical outcomes of muscle-invasive bladder cancer [16], non-small-cell lung cancer [17], inflammatory bowel disease [18], heart failure [19, 20], and coronavirus disease 2019 [21].

Several studies have explored a possible connection between ChE and COPD. For example, a higher concentration of ChE has been shown to originate from the membranes of erythrocytes in COPD patients than in healthy controls [22]. Furthermore, the blood level of ChE is increased in COPD patients, which is also associated positively with levels of interleukin-6 but related negatively to oxidative-damage biomarkers, including advanced oxidation protein products and total protein carbonyls [23]. However, a study to assess the value of the serum ChE level in predicting adverse outcomes in hospitalized patients with AECOPD has not been conducted.

In our hospital, serum ChE levels have been estimated for all hospitalized patients since 2015 as a part of the standard liver function test package, which allowed us to investigate whether ChE levels could predict adverse outcomes in AECOPD patients. Therefore, in this study, we retrospectively collected AECOPD cases admitted to our hospital and their clinical data and then analyzed the relationship between the serum ChE level and hypoxemia severity, hypercapnia, duration of hospital stay (DoHS), and the need for noninvasive ventilation (NIV).

## 2. Methods

### 2.1. Ethical Considerations

The study was conducted in accordance with the Declaration of Helsinki (as revised in 2013). Ethical approval (LW 2022-08; 13 May 2022) of the study protocol was obtained from the Ethics Committee of Wuhu Hospital of Traditional Chinese Medicine (Wuhu, China). The requirement for informed consent was waived due to this being a retrospective study using clinical data.

### 2.2. Participants

We focused on the surviving patients with AECOPD hospitalized at the Wuhu Hospital of Traditional Chinese Medicine from January 2017 to December 2021. Due to low in-hospital mortality in our study, we excluded patients who died during hospitalization. Only the first hospitalization was enrolled in the analysis set for patients hospitalized more than once. The inclusion criteria were as follows: (1) a definitive diagnosis of COPD based on the criteria mentioned in the Global Initiative for Chronic Obstructive Lung Disease guideline published in 2019 [24]; (2) the primary diagnosis upon hospital admission was AECOPD [25]; (3) laboratory examinations (e.g., serum level of ChE, complete blood count, biochemistry, and arterial blood-gas analyses) were carried out by blood tests within 3 days after hospital admission; and (4) patients who survived during hospitalization, including those who were discharged or transferred to the small community hospital. The exclusion criteria were as follows: (1) patients with hepatobiliary disease, malignancy, active tuberculosis infection, or any other chronic infection that may affect the serum ChE level and (2) patients for whom clinical data were not available. The primary indication for using NIV in AECOPD patients is when their PH level falls below 7.35. Other indications encompass hypercapnia, severe hypoxemia that is unresponsive to conventional oxygen therapies, and pronounced breathing difficulties accompanied by respiratory muscle fatigue.

### 2.3. Primary Outcomes

The primary outcomes were hypoxemia severity, hypercapnia, prolonged hospitalization (defined as a hospital stay of >10 days), and NIV requirement.

### 2.4. Data Collection

Data were compiled from the electronic medical record system of the Wuhu Hospital of Traditional Chinese Medicine. Data for the serum level of ChE, C-reactive protein (CRP), and biochemistry (albumin, blood urea nitrogen (BUN), and creatinine) were collected. The serum ChE level was measured using an automatic biochemical analyzer (7020 series; Hitachi, Tokyo, Japan) after the addition of butyrylthiocholine iodide as a substrate. All fasting venous blood samples were collected in the early morning of the day after admission and transported to the Department of Laboratory Medicine for routine testing within 2 hours. The clinical information of AECOPD patients was also collected which included the following: sex; age; medical history (diabetes mellitus, hypertension, coronary heart disease, and heart failure); number of acute exacerbations in the previous 12 months; treatment-related data (whether patients received NIV or invasive mechanical ventilation, whether patients were admitted to the intensive care unit (ICU), and DoHS); results of arterial blood-gas analyses (partial pressure of carbon dioxide (pCO_2_), pH, lactic acid, and oxygenation index (OI)); D-dimer level; and complete blood count (neutrophil-to-lymphocyte ratio (NLR), hematocrit, red blood cell distribution width (RDW), platelet-to-lymphocyte ratio (PLR), and platelet distribution width (PDW)).

### 2.5. Statistical Analyses

Statistical analyses were carried out using SPSS 26.0 (IBM, Armonk, NY, USA) and Prism 8 (GraphPad, San Diego, CA, USA). Data are presented as mean (SD) for variables with a normal distribution, medians (interquartile range (IQR)) for variables with a non-normal distribution, and numbers and constituent ratios for categorical variables. The Mann–Whitney *U*-test was used to compare continuous variables among the two groups. The Kruskal–Wallis test was employed to compare continuous variables among the three groups. Categorical variables were compared using the chi‐squared test or Fisher's exact test. Correlations among the serum levels of ChE, CRP, albumin, and the NLR were analyzed by using Pearson's correlation analysis. We used multivariable logistic regression analysis to evaluate the association of the serum ChE level with NIV requirement and prolonged hospitalization. *p* < 0.05 was considered significant.

## 3. Results

### 3.1. Patient Characteristics at Baseline

**Figure 1:**
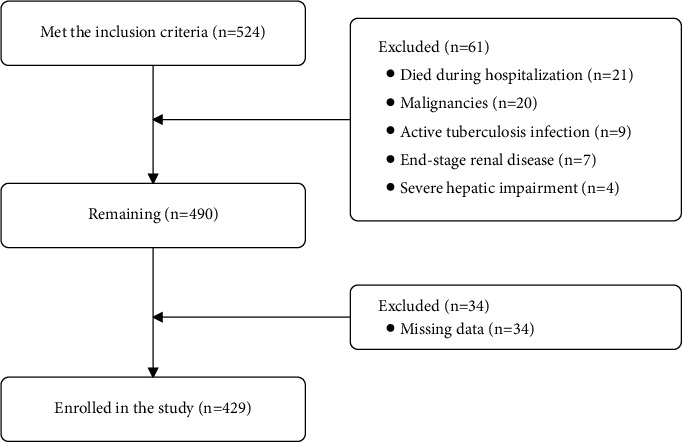
Flowchart of our study.

Patient enrolment is shown in Figure 1. A total of 429 patients hospitalized with AECOPD were enrolled, and 318 of them (74.1%) were men. The median age (IQR) was 76 (range: 70–82) years. A comorbid diagnosis of coronary heart disease was documented in 183 patients (42.7%), whereas 186 (43.4%) were suffering from heart failure. A total of 175 (40.8%) patients presented with hypercapnia. 96 (22.4%) patients received NIV and 18 (4.2%) patients had invasive mechanical ventilation. Thirty-four (7.9%) patients were admitted to the ICU. The data for laboratory examinations (median, (IQR)) were obtained for ChE (5259.00 (4113.50–6757.50) U/L); NLR (6.52 (3.72–12.12)); albumin (37.25 (34.00–40.50) g/L); BUN (6.50 (5.20–9.20) mmol/L); CRP (15.90 (3.85–65.55) mg/mL); OI (286.00 (214.60–345.20)); and pCO2 (44.03 (34.20–59.75) mmHg).

The study population (*n* = 429) was divided into three groups based on their serum ChE levels: “low” (≤4116 U/L; *n* = 143), “moderate” (4116 ‐ 6137 U/L; *n* = 143), and “high” (>6137 U/L; *n* = 143). Patient characteristics at the baseline are shown in Table 1. Compared with patients in the high group for the serum ChE level, those in the low group for the serum ChE level were older; had a higher NLR and RDW; had higher levels of BUN, CRP, and D-dimer; and had a lower eosinophil count, hematocrit, and OI. Also, a higher proportion of patients in the low group for the serum ChE level were admitted to the ICU, had a longer DoHS, and had coexisting heart failure, hypercapnia, and NIV requirement than those in the high group for the serum ChE level.

### 3.2. Association of Clinical Indicators with the Serum ChE Level

**Figure 2:**
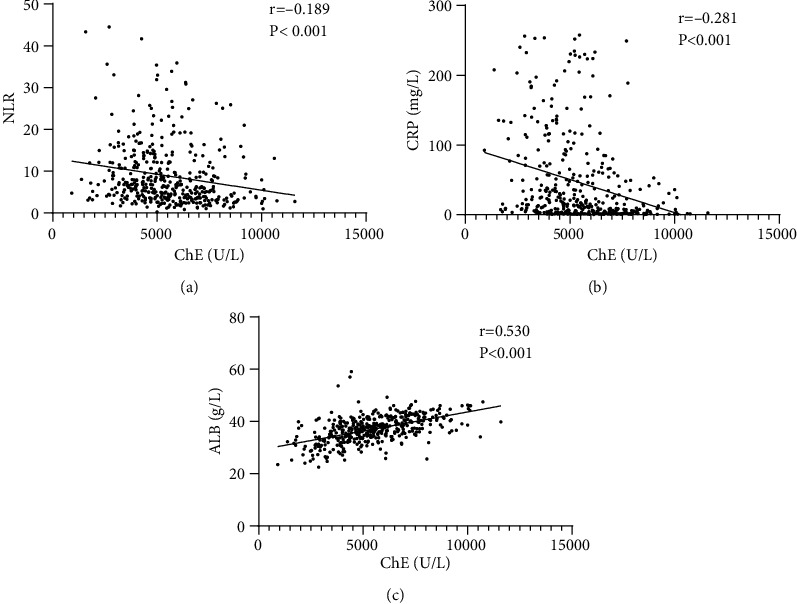
Correlation between the serum ChE level and the NLR level (a), the CRP level (b), and the albumin level (c) in hospitalized patients with AECOPD. ChE: cholinesterase, NLR: neutrophil-to-lymphocyte ratio, CRP: C-reactive protein, and ALB: albumin.

Spearman correlation coefficients showed that the serum ChE level was correlated negatively with the CRP level (*r* = −0.281; *p* < 0.001) (Figure 2(a)) and NLR (*r* = −0.189; *p* < 0.001) (Figure 2(b)). A positive correlation between the serum ChE level and albumin level (*r* = 0.530; *p* < 0.001) is documented in Figure 2(c).

### 3.3. Correlation of the Serum ChE Level with Hypercapnia, NIV Requirement, Hypoxemia Severity, and DoHS

**Figure 3:**
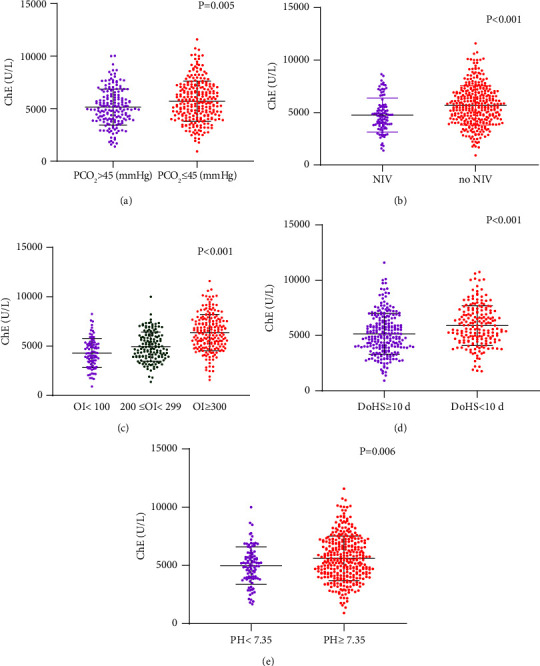
Correlation of the serum ChE level with hypercapnia (a), NIV requirement (b), hypoxemia severity (c), duration of hospital stay (d) and PH (e). ChE: cholinesterase, pCO_2_: partial pressure of carbon dioxide, NIV: noninvasive ventilation, OI: oxygenation index, and DoHS: duration of hospital stay.

A total of 175 (40.8%) patients presented with hypercapnia. The serum ChE level was significantly lower in patients with a pCO_2_ of >45 mmHg than in those with a pCO_2_ of ≤45 mmHg (4980.00 (range, 3972.00–6246.00) vs. 5559.50 (range, 4320.00–7041.50), *p*=0.005) (Figure 3(a)). Ninety-six (22.4%) patients received NIV. The serum ChE level in patients who required NIV was significantly lower than that in patients who did not require NIV (4623.00 (range, 3748.00–5566.00) vs. 5553.00 (range, 4352.00–6881.00), *p* < 0.001) (Figure 3(b)). The number of patients whose OI (mmHg) was >300, between 200 and 300 (contained data for two endpoints), and <200 was 196 (45.7%), 143 (33.3%), and 90 (21%), respectively. The serum ChE level among groups with a high, medium, and low OI showed significant variations (6332.50 (range, 5019.00–7640.00) vs. 4745.00 (range, 3905.00–6065.00) vs. 4285.00 (range, 3246.00–5247.00), *p* < 0.001) (Figure 3(c)). A total of 240 (55.9%) patients stayed in the hospital for ≥10 days and 189 (44.1%) patients stayed for <10 days. The serum ChE level in patients who had a prolonged stay in the hospital was lower than that in patients who had a DoHS of less than 10 days (4905.00 (3910.00–6357.00) vs 5622.00 (4648.50–7149.50), *p* < 0.001) (Figure 3(d)). The number of patients presented with pH < 7.35 was 95 (22.1%). The serum ChE level in patients presented with pH < 7.35 was significantly lower than that in patients presented with pH ≥ 7.35 (4944.00 (range, 3910.00–5944.00) vs. 5466.00 (range, 4252.00–6995.00), *p*=0.003) (Figure 3(e)).

Univariate analysis (Table 2) showed that the serum ChE level, number of acute exacerbations in the previous year, history of heart failure, NLR, hematocrit, RDW, BUN level, and D-dimer level were risk factors for NIV requirement. However, multivariate logistic regression analysis showed that only the serum ChE level of ≤4116 U/L (odds ratio = 2.857, 95% confidence interval = 1.461–5.588, *p*=0.002), number of acute exacerbations in the previous year (1.337, 1.073–1.666, 0.010), and history of heart failure (2.921, 1.731–4.927, <0.001) were associated with the need for NIV (Table 3).

## 4. Discussion

We evaluated the utility of the serum ChE level for predicting adverse clinical outcomes in patients hospitalized with AECOPD, including hypoxemia severity, hypercapnia, DoHS, and the need for NIV. This is the first study focusing on the utility of the serum ChE level for predicting the adverse clinical outcomes of hospitalized patients with AECOPD.

The serum ChE level in patients who needed NIV was significantly lower than that for patients who did not need NIV. The serum ChE level was lower in patients who stayed in the hospital for ≥10 days, had hypercapnia, and an OI of < 200 than patients who stayed in the hospital for <10 days, did not have hypercapnia, and who had an OI of >300, respectively. Multivariate logistic regression analysis revealed a significant association between the serum ChE level and NIV requirement after adjustment for the number of acute exacerbations in the previous year, history of heart failure, NLR, and hematocrit. These findings suggest that the serum ChE level is a favorable predictor of adverse clinical outcomes in patients hospitalized with AECOPD.

Compared with patients who did not require NIV, patients receiving NIV had significantly more acute exacerbations in the previous year, a significantly longer DoHS, and more of them had coexisting heart failure. These data are consistent with the results from previous studies. Crisafulli and colleagues [26] found that patients with a prolonged DoHS had more hospitalizations for AECOPD in the previous year, a worse modified Medical Research Council (mMRC) dyspnea score, and a higher risk of congestive heart failure. Multivariate regression analysis showed that an mMRC dyspnea score of ≥2 and acute respiratory acidosis could be used to predict a prolonged DoHS upon hospital admission. Steriade and coworkers [27] showed that patients with severe acidosis and left-heart dysfunction required prolonged use of NIV when hospitalized with AECOPD. These results could be explained by the following reasons. COPD exacerbation is associated with accelerated loss of lung function [28]. Patients who experience a greater exacerbation burden have worse lung function and a more rapid decline in forced expiratory volume in one second (FEV_1_) and forced vital capacity [29, 30]. Subsequently, a lower FEV % is closely associated with compensated and decompensated respiratory acidosis, which may contribute to increased demand for NIV [31].

We observed that hospitalized AECOPD patients receiving NIV had a higher NLR, RDW, BUN level, and D-dimer level with lower hematocrit and lower OI, as well as higher pH in arterial blood, and pCO_2_. These results illustrated that patients who received NIV were more ill and are concordant with the data from earlier reports. Song and collaborators [32] revealed that the D-dimer level in older patients with AECOPD complicated by respiratory failure was noticeably higher than that in older patients not suffering from respiratory failure. Several studies have reported that increased RDW was strongly associated with poor clinical outcomes, including rehospitalization within 60 days [33] and increased mortality [34]. Karampitsakos and colleagues [35] found patients hospitalized with AECOPD needing NIV presented with increased RDW. Several researchers have reported that the NLR is associated with the severity and exacerbations of COPD [36] and can also be used to predict the prognosis [37]. Yao and colleagues [38] reported that, at a cutoff of 6.24, the sensitivity and specificity of the NLR for predicting in-hospital mortality for AECOPD patients were 81.08% and 69.17%, respectively, with an area under the curve of 0.803. In our study, the median NLR in patients requiring NIV was much higher than 6.24 (i.e., 7.99). This finding suggests that our patients had a greater disease severity.

The BUN level also has a good predictive ability for the prognosis in patients hospitalized with AECOPD. Chen and coworkers [39] found an increased BUN level to be associated with hospital mortality in patients with AECOPD. A BUN level of 7.63 mmol/L could be used as a cutoff for stratification of the critically ill. The median BUN level of patients receiving NIV in our study was 8.3 mmol/L, which is much higher than the cutoff for the research by Chen and colleagues.

Apart from the significant association between the serum ChE level and adverse clinical outcomes in hospitalized AECOPD patients, we also revealed the serum ChE level to be correlated negatively with the CRP level and NLR but correlated positively with the serum albumin level. We speculate that a lower serum ChE level is closely associated with inflammation and malnutrition. Some studies have suggested that the non-neuronal cholinergic system (which is also present in inflammatory cells in the airways) plays an essential part in the onset, development, and progression of chronic inflammatory diseases of the airways, including COPD [40–42]. Acetylcholine exhibits strikingly different expression profiles in proinflammatory and anti-inflammatory genes and is released from different types of inflammatory cells in the airways. ChE helps to hydrolyze acetylcholine, so it is rational to speculate that it has a vital role in COPD pathogenesis. Moreover, the expression and function of cholinoceptors (the most crucial part of the non-neuronal cholinergic system) can be modified by airway inflammation, the drugs used in clinical management, and components in tobacco smoke.

### 4.1. Study Limitations

Our observational study had five main limitations. First, we did not evaluate the predictive value of the serum ChE level in terms of short-term mortality in hospitalized AECOPD patients due to low in-hospital mortality. Second, although patients had a confirmed COPD diagnosis, most were too severely ill to have lung function evaluated during hospitalization, and some patients' baseline lung-function data were overly dated, so we could not consider the impact of lung-function indices. Third, the serum ChE level would be influenced by dietary intake and baseline nutritional status, but such information could not be obtained from these patients. Fourth, this was a retrospective study and carried all the disadvantages and possibilities of biases that such studies have; for example, we could not include the dyspnea score, respiratory rate, heart rate, APACHE II score, and smoking status to assess the patient's disease severity and the baseline conditions comprehensively. Fifth, even though we only included patients whose primary diagnosis upon hospital admission was AECOPD, we were unable to completely rule out concomitant diseases characterized by pulmonary diffusion impairment, including pneumonia, pulmonary embolism, or acute heart failure. Therefore, caution should be taken when interpreting the results.

## 5. Conclusions

The serum ChE level was correlated significantly with complicating severe hypoxemia, hypercapnia, prolonged DoHS, and the need for NIV in patients hospitalized with AECOPD. These findings suggest that the serum ChE level is a clinically important risk-stratification biomarker in patients hospitalized with AECOPD.

## Figures and Tables

**Table 1:** Patient characteristics at baseline stratified by the serum ChE level.

Characteristics	Overall	Low level	Moderate level	High level	*P value*
*Cholinesterase (U/L)*		ChE ≤ 4116	4116 < ChE ≤ 6737	ChE > 6737	
Number of patients	429	143	143	143	
Age	76 (70–82)	78 (73–83)	76 (70–83)	74 (68–81)	0.004
*Sex*					0.465
Male	318 (74.1)	110 (76.9)	107 (74.8)	101 (70.6)	
Female	111 (25.9)	33 (23.1)	36 (25.2)	42 (29.4)	
Number of AEs in the previous year	1.00 (0.00–2.00)	1.0 (0.0–2.0)	1.0 (0.0–2.0)	1.0 (0.0–2.0)	0.935
*Comorbidities*					
Diabetes mellitus (%)	64 (14.9)	13 (9.1)	24 (16.8)	27 (18.9)	0.050
Hypertension (%)	152 (35.4)	48 (33.6)	63 (44.1)	72 (50.3)	0.105
CHD (%)	183 (42.7)	58 (40.6)	47 (33.3)	47 (32.9)	0.314
Heart failure (%)	186 (43.4)	83 (58.0)	65 (45.5)	38 (26.6)	<0.001
*Laboratory examinations*					
NLR	6.52 (3.72–12.12)	7.6 (5.1–13.6)	6.8 (3.9–13.2)	4.71 (2.98–8.73)	<0.001
Hematocrit	39.30 (35.58–43.43)	36.4 (32.5–42.9)	39.2 (36.3–43.1)	41.2 (38.1–43.7)	<0.001
RDW	14.10 (13.10–15.05)	14.6 (13.5–16.2)	14.1 (13.2–14.9)	13.7 (12.9–14.4)	<0.001
PLR	190.74 (117.01–318.08)	220.0 (143.1–363.4)	200.0 (126.6–321.2)	154.3 (100.0–248.4)	0.001
PDW	13.40 (11.80–16.10)	12.9 (11.5–15.3)	13.4 (11.7–16.0)	14.0 (12.0–16.6)	0.035
Albumin (g/L)	37.25 (34.00–40.50)	34.0 (30.7–37.4)	36.8 (34.8–38.7)	40.4 (37.2–42.8)	<0.001
BUN (mmol/L)	6.50 (5.20–9.20)	7.8 (5.2–12.0)	6.2 (5.2–9.0)	6.2 (5.1–7.8)	0.002
Creatinine (mmol/L)	74.70 (59.20–97.10)	75.4 (60.8–119.0)	77.5 (58.3–92.7)	71.3 (58.9–87.1)	0.084
CRP (mg/mL)	15.90 (3.85–65.55)	33.7 (8.6–124.5)	17.3 (4.9–90.4)	5.6 (1.6–26.2)	<0.001
D-dimer (ng/mL)	877.97 (454.15–1654.85)	1250 (840–2520)	870 (520–1680)	561.2 (340.0–890.0)	<0.001
OI	286.00 (214.60–345.20)	243.70 (175.50–292.20)	286.00 (209.20–358.00)	322.80 (280.80–362.00)	<0.001
pCO_2_ (mmHg)	44.03 (34.20–59.75)	47.2 (34.4–65.4)	47.3 (35.3–62.4)	40.6 (32.8–52.7)	0.019
pH	7.40 (7.35–7.44)	7.4 (7.34–7.44)	7.4 (7.34–7.43)	7.41 (7.36–7.44)	0.341
*Outcomes*					
Hypercapnia (%)	175 (40.8)	66 (46.2)	64 (44.8)	45 (31.5)	0.020
NIV (%)	96 (22.4)	47 (32.9)	30 (21.0)	19 (13.3)	<0.001
IMV (%)	18 (4.2)	11 (7.7)	5 (3.5)	2 (1.4)	0.026
ICU (%)	34 (7.9)	20 (14.0)	9 (6.3)	5 (3.5)	0.003
DoHS (days)	10.00 (8.00–14.00)	12 (9–16)	10 (8–13)	9 (7–12)	<0.001

AE, acute exacerbation; CHD, coronary heart disease; NLR, neutrophil-to-lymphocyte ratio; RDW, red blood cell distribution width; PLR, platelet-to-lymphocyte ratio; PDW, platelet distribution width; BUN, blood urea nitrogen; CRP, C-reactive protein; OI, oxygenation index; pCO_2_, partial pressure of carbon dioxide; NIV, noninvasive ventilation; IMV, invasive mechanical ventilation; ICU, intensive care unit; DoHS, duration of hospital stay.

**Table 2:** Characteristics of the patients in the NIV group and the non-NIV group.

Characteristics	NIV	Non-NIV	*Z*/*X*^2^	*P value*
Age	76 (70–81)	76 (70–82)	−0.670	0.503
*Sex*			1.864	0.172
Male (%)	66 (68.7)	252 (75.7)		
Female (%)	30 (31.3)	81 (24.3)		
Number of AEs in the previous year	1.0 (0.0–2.0)	1.0 (0.0–2.0)	−3.366	0.001
*Comorbidities*				
Diabetes mellitus (%)	14 (15)	50 (14.6)	0.011	0.917
Hypertension (%)	38 (39.6)	145 (43.5)	0.478	0.489
Coronary heart disease (%)	36 (35.0)	116 (37.5)	0.196	0.958
Heart failure (%)	66 (68.8)	120 (36)	32.473	<0.001
*Laboratory examinations*				
NLR	7.99 (4.91–14.00)	6.11(3.58–11.33)	−2.711	0.005
Hematocrit	42.7 (38.0–48.5)	38.8 (35.4–42.5)	−4.840	<0.001
RDW	14.7 (13.9–15.8)	13.9 (13.0–14.75)	−4.996	<0.001
PLR	209.3 (131.3–343.4)	188.7 (113.0–308.0)	−1.180	0.238
PDW	13.2 (11.7–15.6)	13.5 (11.8–16.2)	−0.550	0.582
ChE (U/L)	4623 (3748–5566)	5553 (4352–6881)	−4.393	<0.001
ChE level (%)			16.023	<0.001
ChE ≤ 4116 U/L	96 (28.8)	47 (49.0)		
4116 U/L < ChE ≤ 6737 U/L	113 (33.9)	30 (31.3)		
ChE > 6737 U/L	124 (37.2)	19 (19.7)		
Albumin (g/L)	36.7 (33.1–39.9)	37.4 (34.1–40.8)	−1.220	0.222
BUN (mmol/L)	8.3 (6.0–12.3)	6.2 (5.1–8.5)	−3.989	<0.001
Creatinine (mmol/L)	83.0 (53.0–111.8)	73.2 (59.6–92.4)	−1.100	0.270
CRP (mg/mL)	16.4 (5.4–67.3)	15.4 (3.5–62.7)	−0.649	0.516
D-dimer (ng/mL)	1025.0 (647.5–2160.0)	830.0 (435.0–1550.0)	−2.568	0.010
OI	243.05 (160.75–299.03)	302.00 (232.40–351.75)	−4.959	<0.001
*OI level*			30.157	<0.001
≥300	24 (25)	172 (52.1)		
200–299	36 (37.5)	107 (32.4)		
<200	36 (37.5)	51 (15.5)		
pCO_2_ (mmHg)	68.9 (55.0–81.0)	40.2 (32.2–51.4)	−10.740	<0.001
pH	7.35 (7.28–7.41)	7.41 (7.37–7.44)	−6.718	<0.001
*Outcomes*				
Hypercapnia (%)	83 (86.5)	92 (27.6)	106.785	<0.001
IMV (%)	13 (13.5)	5 (1.5)	26.873	<0.001
ICU (%)	27 (79.4)	7 (20.6)	69.153	<0.001
DoHS (days)	12.0 (9.0–16.0)	10.0 (8.0–13.0)	−3.253	0.001

AE, acute exacerbation; CHD, coronary heart disease; NLR, neutrophil-to-lymphocyte ratio; RDW, red blood cell distribution width; PLR, platelet-to-lymphocyte ratio; PDW, platelet distribution width; BUN, blood urea nitrogen; CRP, C-reactive protein; OI, oxygenation index; pCO_2_, partial pressure of carbon dioxide; NIV, noninvasive ventilation; IMV, invasive mechanical ventilation; ICU, intensive care unit; DoHS, duration of hospital stay.

**Table 3:** Logistic regression analyses of the risk factors associated with NIV requirement in hospitalized AECOPD patients.

Variables	*B*	SE	Wald	*P value*	Exp (*B*)	95% CI for EXP (*B*)	
						Lower	Upper
Number of AEs in the previous year	0.290	0.112	6.682	0.010	1.337	1.073	1.666
Cholinesterase (U/L)			10.491	0.005			
4116 < ChE ≤ 6737	0.393	0.348	1.277	0.258	1.481	0.749	2.929
ChE ≤ 4116	1.050	0.342	9.416	0.002	2.857	1.461	5.588
Hematocrit	0.032	0.017	3.403	0.065	1.032	0.998	1.068
NLR	0.031	0.016	3.627	0.057	1.031	0.999	1.064
History of heart failure	1.072	0.267	16.138	<0.001	2.921	1.731	4.927
Constant	−4.275	0.789	29.332	<0.001	0.014		

AE, acute exacerbation; NLR, neutrophil-to-lymphocyte ratio.

## Data Availability

The data that support the findings of this study are available from the corresponding author upon request.
